# Unveiling the Multifaceted Neuroprotection of Dihydroxytrimethoxyflavone: Memory Enhancement Beyond Cholinergic Boost

**DOI:** 10.1002/alz.089173

**Published:** 2025-01-09

**Authors:** Varinder Singh, Manjinder Singh, Richa Shri

**Affiliations:** ^1^ Department of Pharmaceutical Sciences and Technology, Maharaja Ranjit Singh Punjab Technical University, Bathinda, Punjab India; ^2^ Chitkara College of Pharmacy, Chitkara University, Rajpura, Punjab India; ^3^ Punjabi University, Patiala, Punjab India

## Abstract

**Background:**

Several studies have shown the influential role of nutraceuticals on cognition and mental functions. Dihydroxytrimethoxyflavone, a natural flavone found in herbal drugs, is documented to be neuroprotective in different model systems. Nevertheless, possible memory improvement effects of dihydroxytrimethoxyflavone via nuclear factor‐E2‐related factor 2 (Nrf2) (a crucial regulator of antioxidative system) has not been systematically evaluated. Thus, the present study was aimed to investigate the memory improvement effects of dihydroxytrimethoxyflavone in mouse model of lead induced memory impairment and to reveal the possible molecular mechanism involving Nrf2, oxidative stress, cholinergic signalling and beta amyloid.

**Method:**

Memory impairment in mice was induced by administering lead acetate for 28 days. Dihydroxytrimethoxyflavone (5‐10 mg/kg, p.o.) was administered daily to animals 60 min prior to lead acetate for 28 days. Memory functions (Morris Water Maze), brain acetylcholinesterase (AChE) activity, oxidative stress, beta amyloid and histopathological studies (H&E and congo red staining for beta amyloid) were determined to measure memory improvement effects of dihydroxytrimethoxyflavone.

**Result:**

Dihydroxytrimethoxyflavone showed improvement in memory of animals. It reduced acetylcholinesterase activity and beta amyloid levels. Further, dihydroxytrimethoxyflavone could significantly increase antioxidant enzymes, Nrf2 expression and its nuclear translocation as well as its downstream antioxidant protein expression, NAD(P)H/quinone oxidoreductase 1 and heme oxygenase‐1.

**Conclusion:**

Dihydroxytrimethoxyflavone improved memory in animals via activating Nrf2‐mediated antioxidant pathway, increasing antioxidant defence and inhibiting and amyloid protein burden. Thus, dihydroxytrimethoxyflavone may have beneficial effects in lowering the onset of dementia.

